# Public Perceptions and Willingness-to-Pay for Nanopesticides

**DOI:** 10.3390/nano12081292

**Published:** 2022-04-11

**Authors:** Peiyuan Liu, Xiaodong Zheng, Shuangyue Shangguan, Lina Zhao, Xiangming Fang, Yuxiong Huang, Slav W. Hermanowicz

**Affiliations:** 1 Tsinghua-Berkeley Shenzhen Institute, Shenzhen International Graduate School, Tsinghua University, Shenzhen 518055, China; peiyuan.liu@outlook.com (P.L.); hermanowicz@ceeucb.com (S.W.H.); 2 School of Economics, Zhejiang Gongshang University, Hangzhou 310018, China; zhengxd@cau.edu.cn; 3 College of Economics and Management, China Agricultural University, Beijing 100083, China; 18811763751@163.com; 4 Institute for Hospital Management, Shenzhen International Graduate School, Tsinghua University, Shenzhen 518055, China; zln20@mails.tsinghua.edu.cn; 5 Department of Civil and Environmental Engineering, University of California, Berkeley, CA 94720, USA

**Keywords:** nanopesticides, public perception, willingness-to-pay, interval regression model, survey

## Abstract

The usage of pesticides is deemed essential to ensure crop production for global food security. Conventional chemical pesticides have significant effects on ecosystems. Nanopesticides are increasingly considered an emerging alternative due to their higher efficiency and lower environmental impacts. However, large knowledge gaps exist in the public perceptions and willingness-to-pay (WTP) for nanopesticides. Thus, we conducted a regional survey of pesticide users and food consumers on perceptions and WTP for nanopesticides across China. We found that 97.4% pesticide users were willing to pay for nanopesticides, with a main price from 25% to 40% higher than for conventional pesticides. Experience with applying pesticides, income, familiarity with and attitude toward nanopesticides, and trust in industries were significant determinants of WTP. Although the public were not familiar with nanopesticides, they had positive attitudes toward their future development and supported labeling nanoscale ingredients on products. Pesticide users presented high trust levels in governments and industries, while 34% of food consumers neutrally or distrusted industries in selling and production. This study highlights the socioeconomic and technological aspects of nanopesticides, which could provide guidance for industries to develop market strategies and for governments to design relevant regulation policies effectively, contributing to crop yield improvement and sustainable agriculture.

## 1. Introduction

The rapid growth of the global population, which is predicted to reach nearly 9.8 billion people by 2050, requires food production to increase by 50% compared to the levels in 2012 [1]. However, crop production is significantly suppressed by biotic stresses, such as pests, weeds, and diseases caused by fungi, bacteria, and viruses [2,3]. Application of pesticides is a critical way to mitigate these biotic stresses [2]. Although conventional chemical pesticides are effective, they simultaneously pose severe threats to the ecosystem [3,4]. Specifically, due to the low efficiency of conventional pesticides (~90% lost to environment) [5], farmers must increase the application frequency and amount to achieve better crop yields, resulting in 2 million tons per year of conventional pesticides applied worldwide [1]. Such extensive use not only aggravates environmental contamination (e.g., anoxic water bodies, loss of biodiversity, and ecotoxicity) [5,6], but also poses risks to public health directly and indirectly [7,8]. In addition, the long-term use of conventional pesticides induced resistant crop varieties [9] and increased farmers’ economic costs [10]. Therefore, a revolution of pesticides is urgently needed to improve crop production and maintain sustainability.

With the rapid development of nanotechnologies, nanopesticides have been increasingly anticipated for the agrochemical sector, including nanoemulsions, nanocapsules (e.g., with polymers), and inorganic engineered nanoparticles (ENPs, for example, metals, metal oxides, and nanoclays) [11]. Composed of nanoscale active ingredients (AIs), nanopesticides exhibited broad-spectrum insecticidal, fungicidal, and herbicidal properties [3,12]. Furthermore, nanopesticides can enhance solubility, control release, increase leaf adhesion, and improve the stability of AIs [13], resulting in elevated efficacy and durability, as well as the reduction in applied AIs [14]. Thus, the application of nanopesticides can maintain or increase crop yields with lower application rates, which would potentially minimize the risks to ecosystems [15]. In addition, the global market for pesticides is estimated to grow from US$75 billion in 2013 to US$90 billion by 2023 [16], and nanopesticides have the potential to result in multibillion-dollar benefits [5]. Nevertheless, only a few commercial products among synthesized nanopesticides have been commercialized (e.g., Kocide 3000 (Dupont), and AZteroid FC (Vive Crop Protection)), as a result of three major barriers to technology readiness and implementation (i.e., efficient delivery at the field scale, regulation and safety concerns, and consumer acceptance) [16].

Currently, research on nanopesticides has been mainly focused on their performance [15], mechanisms [17], environmental fate [2], and ecosystem implications [18,19]. However, huge knowledge gaps exist related to nano-governance and socio-economic aspects, particularly in public perceptions and willingness-to-pay (WTP) for nanopesticides, which are essential to promote the usage and market share of nanopesticides [15]. Specifically, the perception of familiarity with nanotechnology could have a positive impact on risk and benefit perceptions, which would further affect attitudes toward and the acceptance of nanotechnology [20]. In particular, WTP, which is defined as the maximum price that a consumer would accept to purchase one unit of a product or service [21], could shape the direction of the marketing price and enable stakeholders to better match the needs and desires of the public.

Factors that could affect the public perceptions and WTP of nanotechnologies or emerging agri-food technologies depend on complicated mechanisms and multiple aspects. For example, socio-demographic factors (i.e., age, gender, education, and income) were shown to be related to understanding the risks and benefits of nanotechnologies [22,23]. Familiarity with [24] and attitude toward [25] novel agri-food technologies had implications for the willingness to purchase these technologies (e.g., nano-enabled food packaging or ingredients). On the other hand, the safety of nanotechnology applications has been questioned in the past few years, resulting in increased requirements for nanotechnology labeling [26], which could help the public manage risks and benefits while purchasing [27,28,29]. Another significant aspect of public perceptions and WTP is social trust, which could have an impact on the risk perceptions and communication of new technologies by building social relationships [20]. Multiple studies have shown that the acceptance of nano-enabled products was greatly influenced by trust in industries [30]. Trust in governments to manage risks was also a major concern when assessing nanotechnology applications [31]. There has also been uncertainty regarding the experience of applying pesticides, which could affect farmers’ acceptance of new-type pesticides [32]. Users may not want to change their habits if they have used conventional pesticides for a long period time [32]. However, they were more likely to accept new pesticides after realizing the long-term hazards of conventional chemical pesticides [32]. Hence, it is essential to understand the multiple factors influencing public WTP for nanopesticides, which are currently missing in available studies.

To investigate public perceptions of and WTP for nanopesticides, we conducted a regional survey of pesticide users and food consumers across China, with almost 400 valid responses. China, as a large agricultural country with 23.6% of its workforce employed in the agricultural sector in 2020 [33], is the largest consumer of pesticides in the world [34]. China has made impressive progress, taking 9% of the planet’s arable land to feed 22% of the worlds’ population [35]. However, as a result of its ongoing urbanization, population growth, and severe environmental impacts, China is facing new challenges to sustainable agriculture [35]. As nano-enabled agriculture has exhibited potential to address these challenges, it is worthwhile to investigate pesticide users and food consumers in China as a representative case study on nanopesticides to explore future agricultural development. The objectives of this study were to (1) examine the price ranges of public WTP for nanopesticides; (2) identify the factors influencing pesticide users’ WTP for nanopesticides; (3) estimate the WTP for nanopesticides under different pesticide user profiles; and (4) explore general public perspectives on nanopesticides. In this study, we combined the advantages of multiple models (i.e., the Heckman model, interval regression model, ordinary square least model, and ordered logistic model) to estimate statistical outcomes. Our findings could narrow the gaps among academia, the public, industries, and governments, thereby helping to assess the market potential, facilitate research and development, and design regulation policies for nanopesticides. This research further aimed at meeting the increasing demands in food production and making agriculture more sustainable.

## 2. Materials and Methods

### 2.1. Data Collection

**Figure A1:**
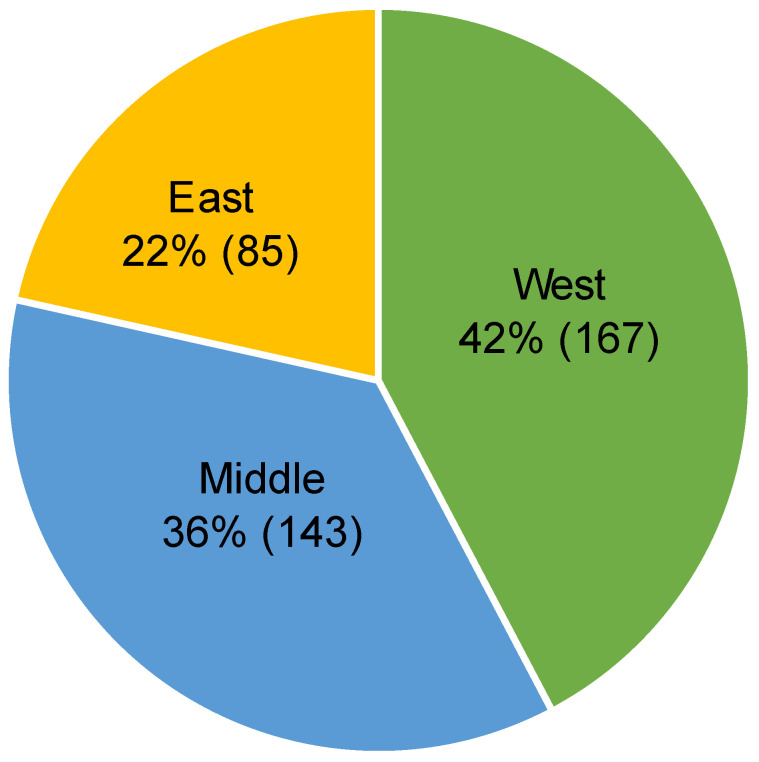
The distribution of sampling sites in the western, middle, and eastern parts of China. Numbers in the parentheses represent the sample size of regions. Eastern part: Fujian province (16), Jiangsu province (24), Zhejiang province (11), Hainan province (18), Shanghai (16). Middle part: Hubei province (42), Hunan province (19), Anhui province (32), Jiangxi province (50). Western part: Yunnan province (24), Sichuan province (17), Guizhou province (27), Chongqing (59), Tibet autonomous region (15), Guangxi autonomous region (25).

The survey was conducted between 24 July 2020 and 5 August 2020 via face-to-face interviews with a questionnaire. We stratified the survey sites into the western, middle, and eastern parts of China. The locations included the countryside located in two municipalities directly under the central government (i.e., Chongqing and Shanghai), eight cities of two autonomous regions (i.e., Guangxi and Tibet) and 56 cities in 11 provinces (details in Figure A1 of Appendix A). The respondents were randomly chosen and we collected 395 fully completed surveys. The survey included 232 pesticide users (i.e., farmers using pesticides) and 163 food consumers (i.e., people from aquaculture and animal husbandry not using pesticides). Eighteen surveys were not completed and were discarded in the following analysis.

More specifically, the appropriate sample size was estimated before investigation by calculating the equation of simple random sampling with substitution [36,37]:

(1) $$N=\frac{4{\left(Z_{crit}\right)}^{2}p\left(1-p\right)}{D^{2}}$$

where *N* is the sample size; *Z_crit_* is the standard normal deviation corresponding to the selected confidence level (CI); *D* is the minimum expected difference, which is specified here subjectively to reflect the difference between the upper and lower limit of an expected CI (i.e., the total width of the expected CI); and *p* is a pre-study estimate of the proportion to be measured. We set the CI at 95 percent that yielded *Z_crit_* = 1.960, assumed *D* = 10% (0.1), and estimated *p* = 0.9 (using the proportion from a preliminary survey on pesticide users’ willingness-to-pay for nanopesticides; approximately 90% replied “yes”). Based on these assumptions, Equation (1) yielded a sample size of *N* = 138. Therefore, considering possible invalid responses, we expanded the survey scale and the final 232 valid responses of pesticide users met the requirements of the sample size.

### 2.2. Questionnaire and Measurements

#### 2.2.1. Variable Selection

Ten independent variables were selected to evaluate public acceptance of emerging technologies. Factors related to socio-demographic information (i.e., gender, age, education, and income) and public perceptions of nanopesticides (i.e., familiarity with and attitude toward nanopesticides, labeling preference, and trust in governments and industries) were investigated. In addition, we included experience of applying pesticides and the associated quadratic term to examine the possible incremental or diminishing effects.

**Figure A2:**
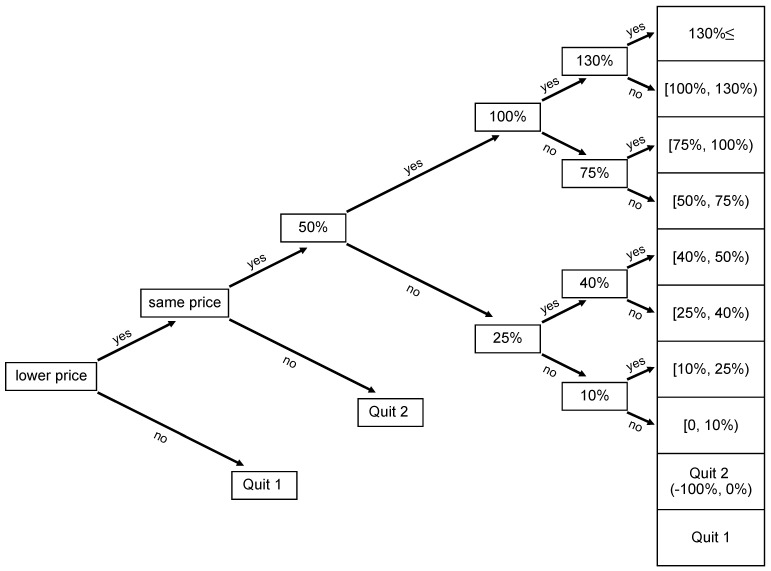
Sequence of questions about the willingness-to-pay for nanopesticides. At each node, respondents were asked whether they would be willing to purchase nanopesticides if the price was lower, the same as, or X% higher than conventional pesticides. The right column shows bounds on the percentages that respondents were willing to pay for nanopesticides above that for conventional pesticides.

The dependent variables included (a) the decision to spend money on nanopesticides when the price was lower than that of conventional pesticides. If respondents indicated unwillingness, they would end the questionnaire. Otherwise, respondents were asked specific follow-up questions about (b) the price ranges of WTP for nanopesticides (Figure A2 in Appendix A).

#### 2.2.2. Questionnaire Design

**Figure 1:**
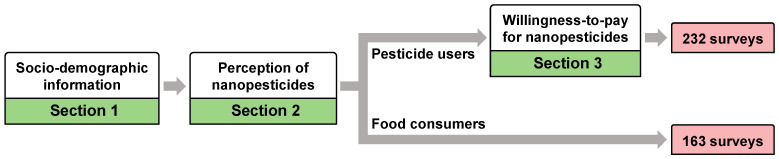
Flowchart for the questionnaire survey.

As illustrated in Figure 1, the questionnaire incorporated 12 questions that were divided into three sections; the complete questionnaire is presented in Appendix B. The first section included four socio-demographic questions (i.e., gender, age, education, and income), followed by five questions relevant to the perceptions of nanopesticides (i.e., familiarity with and attitude toward nanopesticides, labeling preference, and trust in governments and industries) in the second section. Respondents were then asked whether they planted crops needing pesticides. The food consumers (i.e., people from aquaculture and animal husbandry) would not need to purchase pesticides and quitted the survey. Only pesticide users (i.e., farmers) continued with the third-section questions, including the experience of applying pesticides and the WTP for nanopesticides (Figure 1).

The WTP questions were designed using the contingent valuation method (CVM), a mature tool used to estimate public WTP for environmental goods and services in the marketplace [21,38], and widely applied in the sectors of foods and pesticides [39,40,41,42]. The CVM-based questionnaire is typically framed as an open-ended question, such as “how much money you would be willing to pay for the target goods or services?” or as a “yes/no” question that determines whether or not the respondent would be willing to pay $X for the target goods or services [43]. We chose the doubled-bounded CVM to ask a series of questions to progressively narrow down each respondent’s bounds on WTP, resulting in nine intervals (in %) that consumers would be willing to pay for nanopesticides per kilogram over that for conventional pesticides per kilogram: −100–0%, 0–10%, 10–25%, 25–40%, 40–50%, 50–75%, 75–100%, 100–130%, and ≥130% (Figure A2 in Appendix A). Compared with point data by asking a single open-ended or yes/no question, intervals can generate more efficient estimations and be closer to reality by avoiding the randomness of respondent answers [44,45].

### 2.3. Data Analysis

The survey data were analyzed using the Stata programming software. A descriptive statistical analysis of 232 pesticide users was conducted. In order to explore the influencing factors of WTP for nanopesticides, the Heckman model was firstly used to test whether there would be sample selection bias [46] if we excluded the six samples who missed the specific price ranges of WTP (i.e., they would not like to spend money on nanopesticides even at a lower price, as Quit 1 shown in Figure A2 in Appendix A).

#### 2.3.1. Theory of the Heckman Model

Sample selection bias may arise when values of dependent variables are missing or unobserved caused by another process (e.g., self-selection by individuals or data units investigated, sample selection decisions by analysts or data processers) [46,47]. For example, if the appearance of outcome variable *y_i_* depends on a selection variable *z_i_*, such incidental truncation may result in a missing data problem of *y_i_* and biased coefficient estimation using standard regression techniques (e.g., OLS). In order to resolve this potential bias, the Heckman model was introduced and assumed a two-stage relationship (Equations (A1)–(A3) in Appendix A). The first step in this model is to determine whether an observation in an overall population appears in the final representative samples, and the second step is to model the relationship between the dependent and independent variables in the final selected samples [46]. With the maximum likelihood estimation in the Heckman model, rho (ρ; the correlation between error terms in the selection and outcome equations) could be examined to indicate whether or not sample selection bias exists [46]. If rho is significant, traditional techniques (e.g., OLS) would report biased β estimation. In this situation, the results of the Heckman model can provide consistent and asymptotically efficient estimates by correcting selection bias [48]. Otherwise, traditional regression methods could generate efficient estimates by using selected samples. More details on the Heckman model were provided in Appendix A.

#### 2.3.2. Interval Regression Model

To further examine the significance levels of different independent variables for 226 samples, the interval regression model was used as a preferred method when the outcome was measured as interval data, left-censored data, or right-censored data [48,49]. Other models (i.e., the ordinary least squares (OLS) model and ordered logistic model) were not chosen due to limitations. Specifically, the OLS model would use the interval medians as a dependent variable’s values and use the upper or lower limit values for left-censored data or right-censored data, which neglects the uncertainty distribution of the dependent variable and reduces the accuracy of the results [50]. In addition, the ordered logistic model would order intervals sequentially as dependent variable’s values, which does not take the threshold values into account and results in a loss of information within the dependent variable [49].

By using the interval regression model, we assumed that each respondent *i* had a WTP for nanopesticides $Y_{i}^{*}$ that was related to independent variables $X_{i}$ in the following way:

(2) $$Y_{i}^{*}=X_{i}\beta +\varepsilon_{i}$$

where $\varepsilon_{i}$ was assumed to be a normally distributed term with zero mean [48].

We did not observe $Y_{i}^{*}$ directly, but we knew it fell within some interval [$Y_{i1}$, $Y_{i2}$] based on the responses from a series of double-bounded CVM questions (Figure A2 in Appendix A). Therefore, the likelihood contribution of respondent *i* was $\mathrm{Pr}\left(Y_{i1}\leq Y_{i}^{*}\leq Y_{i2}\right)$ or $\mathrm{Pr}\left(Y_{i1}\leq X_{i}\beta +\varepsilon_{i}\leq Y_{i2}\right)$. For left-censored data (the unobserved $Y_{i}^{*}$ was less than or equal to a fixed upper endpoint) and right-censored data (the unobserved $Y_{i}^{*}$ was greater than or equal to a fixed lower endpoint), the likelihood contributions were $\mathrm{Pr}\left(X_{i}\beta +\varepsilon_{i}\leq Y_{i2}\right)$ and $\mathrm{Pr}\left(Y_{i1}\leq X_{i}\beta +\varepsilon_{i}\right)$, respectively. The maximum likelihood function was estimated using the command *intreg* in Stata, and the specific Equations (A4)–(A8) were illustrated in Appendix A.

#### 2.3.3. Robustness Test

The ordinary least squares (OLS) and ordered logistic models were used to identify the robustness and credibility of the interval regression model. Specifically, we converted the interval data, left-censored data, and right-censored data of WTP into point data of the interval median, upper limit value, and lower limit value, respectively, to estimate the OLS regression model. Meanwhile, nine price ranges of WTP (Figure A2 in Appendix A) were converted into ordinal numbers 1–9 sequentially for the ordered logistic model using maximum likelihood estimation. The independent variables in the OLS and ordered logistic models remained unchanged with that in the interval regression model.

In addition, the relative influence importance of different variables was compared using standard beta coefficients. The plots of the public’s perspectives on nanopesticides were created using the online OmicShare Tools [51].

## 3. Results and Discussion

### 3.1. Descriptive Statistics of Variables

Among all 395 samples, 163 food consumers answered the survey, except for questions regarding the experience of applying pesticides and the WTP for nanopesticides. A total of 232 pesticide users responded to all the questions. The descriptive statistics of the pesticide users were summarized in Table 1.

As shown in Table 1, the 232 pesticide users were between 25 and 75 years old (median = 46), and 17.2% were female while 82.8% were male. Overall, the participants were educated and had an average 11-year education level (mean = 11.1, median = 12). There was a significant range in annual incomes (standard deviation = 15.7), with the median level at 130,000 RMB (approximately 20,000 USD). The participants’ average experience in applying pesticides was more than 15 years, and the maximum was 52 years. Although the current level of familiarity with nanopesticides was low (mean = 2.6), pesticide users had relatively supportive attitudes toward the future development of nanopesticides (mean = 4, median = 4). For labeling indications, the participants generally preferred to be informed that the product contains nano-components (mean = 4.2, minimum = 3). Pesticide users strongly trusted governments and industries regarding supervision, production, and selling (medians = 4).

**Figure 2:**
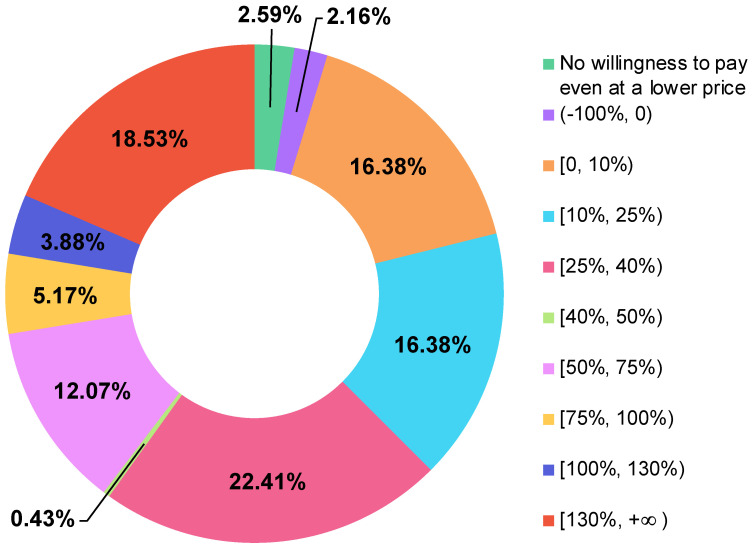
The distribution of the price ranges (% of WTP for nanopesticides over that for conventional pesticides).

Not surprisingly, based on the above positive attitudes, most pesticide users (97.41%) were willing to spend money on nanopesticides. Only six pesticide users (2.59%) would not like to spend any money on nanopesticides, even if the price was lower than that of conventional pesticides (Table 1 and Figure 2). The high proportion of WTP for nanopesticides was much higher than that of WTP for other nano-enabled food products. For example, almost 50% of consumers refused to purchase foods (e.g., canola oil) with nano-packaging, nanodrop, and nano-sensor attributes [52]. The distinct proportions of WTP for different nanoproducts could result from various survey subjects; particularly, the more directly consumers were in contact with nanoproducts, the less likely they were willing to use nanoproducts [53]. Compared to nanopesticide users, fewer food consumers were willing to purchase foods engaged with nanotechnology. This high public purchase intention of nanopesticides could motivate academia, industries, and governments to advance the research and development of nanopesticides, rather than being impeded by worries and uncertainties about public rejection. Moreover, as illustrated in Figure 2, 2.16% of pesticide users would be willing to purchase nanopesticides only if the price was lower than that of conventional pesticides. The main price range that respondents were willing to pay for nanopesticides was 25–40% higher than that of conventional pesticides (Figure 2), guiding industries to improve market strategies and price nanopesticides more appropriately in the future.

### 3.2. Sample Selection Bias and Model Robustness Evaluation

As shown in Figure 2, six pesticide users were not willing to pay for nanopesticides even if the price was lower than that of conventional pesticides, which led to the missing data for the price range. The Heckman model was used to evaluate the sampling selection bias, and we confirmed that these six samples could be excluded as the value of rho (Table A1 in Appendix A) in the Heckman model was not significant [46]. Therefore, the interval regression model on 226 samples was subsequently utilized to evaluate the factors influencing WTP for nanopesticides (Table 2), with detailed discussion in the following section. In order to enhance the credibility of the results, OLS and ordered logistic models were used to verify the robustness of the interval regression model (Table 2). The coefficients of the OLS model were aligned well with those of the interval regression model (Table 2). No obvious differences in the significance levels of variables were found among the OLS, ordered logistic, and interval regression models (Table 2), indicating good reliability of the results obtained by the interval regression model.

### 3.3. Determinants of Willingness-to-Pay for Nanopesticides

As shown in Table 2, both the experience of applying pesticides and the associated quadratic term were statistically significant. There were diminishing and incremental trends before and after 27-year experience, which was a relatively intermediate-level of experience for pesticide users. Early career and richer-experience pesticide users reported higher WTP price ranges than intermediate-experience pesticide users. Specifically, compared with early career pesticide users, intermediate-experience pesticide users would not like to change their habits to adapt to the new routine as they have already formed usage patterns with the conventional pesticides [32]. Meanwhile, the rich-experience pesticide users would try emerging alternatives with higher efficiency and better sustainability after realizing the long-term hazards of conventional chemical pesticides [32].

On the other hand, pesticide users with higher income would be willing to pay more for nanopesticides compared to individuals with lower income. For every 100,000 RMB (approximately 15,385 USD) increase in annual income, the WTP price range would increase by 1.39% (Table 2), which was attributed to greater purchasing power [32]. Furthermore, income was positively correlated with risk preference [54], and risk-takers were more prone to invest in emerging alternative technologies [55,56]. Therefore, in the early stage of promoting nanopesticides, industries could target main markets to regions with better economic situations, and pesticide users with junior and rich experience.

Familiarity with and attitude toward nanopesticides were found to positively and significantly influence the price range of WTP for nanopesticides (Table 2). A sense of familiarity can be created by a generally positive framing of nanotechnology in the media, which could mitigate consumers’ negative responses to risky content, while positive beliefs may confirm benefit information [57]. Moreover, people with low familiarity may be initially less interested in emerging alternatives [57]. It would be important to use media exposure (e.g., science-related news and education programs) and interpersonal communication in an elaborative manner (e.g., lectures) to strengthen perceived familiarity [58]. Increased familiarity would lead people to have a more favorable attitude toward technology [59], further facilitating the acceptance of nanopesticides.

In addition, the price range of WTP for nanopesticides could be notably improved with the rise of the trust level in industries (Table 2). General social trust in the food industry can evoke the preference for emerging nanotechnology products [60,61]. Therefore, maintaining a good corporate reputation would play a key role in marketing nanopesticides. Industries are supposed to operate in accordance with laws and regulations and to carry out effective public-relations strategies simultaneously (e.g., media advertisements, posters, and proactive dialog between different stakeholders through workshops or forums). While trust in governments did not significantly influence pesticide users’ WTP for nanopesticides (Table 2), governments have responsibilities in transferring relevant knowledge to the public (e.g., through training programs for farmers to learn practice techniques and the benefits and risks of nanopesticides) and developing regulations (e.g., registration of nanopesticides, legality of industries, and use and recycling management).

Gender, age, and education level were not significant determinants of WTP. Although labeling preference was also not a significant influencing factor of pesticide users’ WTP for nanopesticides (Table 2), the participants generally agreed that product labels must indicate the usage of nanocomponents (Table 1). Such labeling would not only increase public familiarity with nanotechnology, but also be beneficial for consumers who want to avoid risks, in addition to those who aim to benefit from nanotechnology [57]. However, labeling alone is insufficient to educate the public [28], and comprehensive knowledge of nanopesticides should also be provided.

The standardized beta coefficients in the regression model were further calculated to examine which variables contributed most to the interval regression model (Table A2 in Appendix A), with a higher absolute value indicating a stronger influencing effect of the corresponding independent variable [62]. The experience of applying pesticides was found to have a greater influence on public WTP for nanopesticides than the other variables. Familiarity, trust in industries, attitude, and income had similar influencing importance. These results were based on statistical regressions, which may be different in reality. Overall, these significant determinants of WTP for nanopesticides could provide a direction for industries about which group of pesticide users would be the target customers (e.g., people with high income and high familiarity) and also indicate to policy-makers how they can influence the public acceptance of nanopesticides (e.g., by improving public familiarity and strengthening regulations to increase trust levels in industries).

### 3.4. Estimations of Willingness-to-Pay for Distinct Consumer Profiles

Based on the results of the interval regression model, we estimated the actual WTP for distinct pesticide users’ profiles (Table A3 in Appendix A). For example, pesticide users with 23-year experience in applying pesticides who were a little unfamiliar with nanopesticides and completely distrusted industries, would be willing to pay prices 1% lower for nanopesticides than that for conventional pesticides. In contrast, pesticide users with 13-year experience alongside general familiarity with nanopesticides and a neutral trust level in industries, would be willing to pay 47% more for nanopesticides. Table A3 also illustrates that pesticide users with 5-year experience in applying pesticides, who were very familiar with nanopesticides and strongly trusted industries, would be willing to pay 112% more for nanopesticides.

### 3.5. General Public Perspectives on Nanopesticides

**Figure 3:**
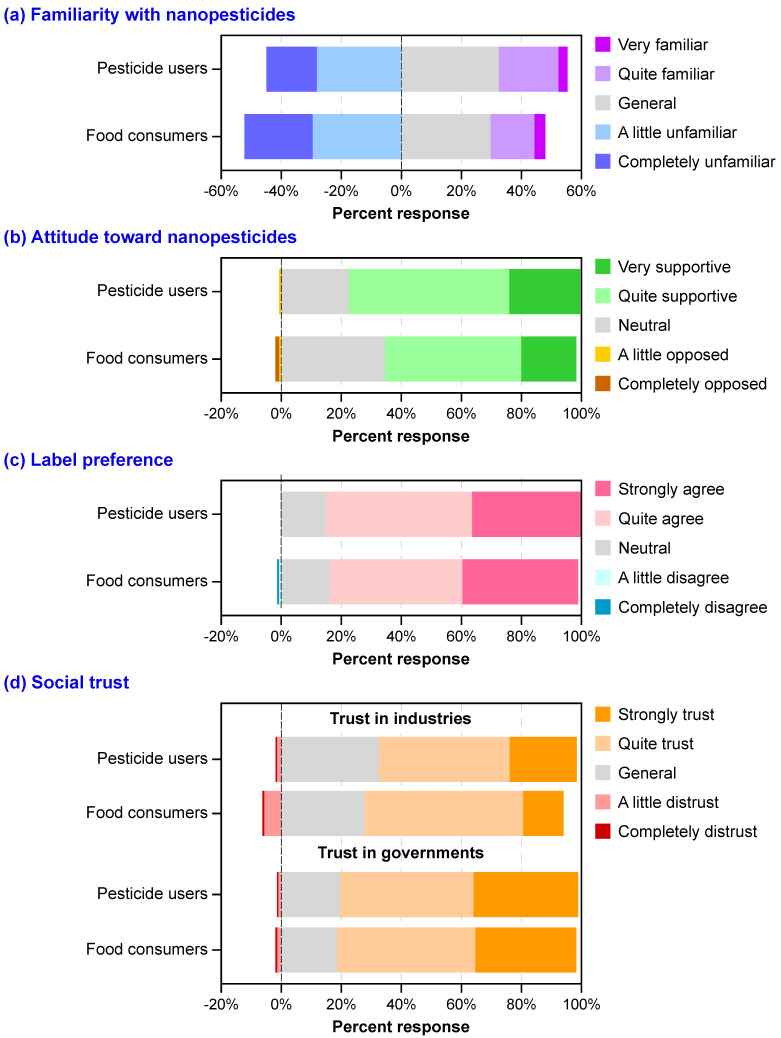
Comparison of public’s perspectives on nanopesticides between pesticide users (i.e., farmers) and food consumers (i.e., people from aquaculture and animal husbandry). Public responses when asked (**a**) “How familiar are you with nanopesticides?”; (**b**) “What is your attitude toward the future development of nanopesticides?”; (**c**) “Do you agree that the product label of nanopesticides must indicate that it contains nano-components?”; (**d**) “Do you trust that industries (manufactures and retailers) could produce and sell nanopesticides legally?” and “Do you trust that governments could supervise the safety risks of nanopesticides?”.

As discussed above, 163 food consumers (i.e., those in aquaculture and animal husbandry who did not use pesticides) also participated in the survey. We analyzed all responders’ (i.e., 163 food consumers and 232 pesticide users) perspectives on nanopesticides to assess the overall perceptions (Figure 3).

In general, nearly half of the survey participants were not familiar with nanopesticides (see light blue and dark boxes in Figure 3a), which was consistent with the results of various surveys that indicated the knowledge of food-relevant nanotechnologies in the general population was low [63]. Food consumers had a lower familiarity level with nanopesticides than pesticide users (Figure 3a). Nevertheless, few people in both groups opposed the future development of nanopesticides (Figure 3b). It implied that there are significant expectations regarding nanopesticides, which have the potential to be highly accepted in the market. In addition, most of the public had neutral positions or agreed that products should have labeling indications for nanocomponents (Figure 3c). The requirement for labeling indications should be incorporated into regulations by governments. Although the general public highly trusted governments, 6% of food consumers distrusted industries, and 28% of food consumers had general trust levels in industries (Figure 3d). Industries producing and selling nanopesticides need to put effort into communicating not only with pesticide users but also with food consumers to enhance general social trust. Otherwise, food consumers would not purchase the nanopesticide-engaged foods, which would further negatively influence pesticide users’ WTP for nanopesticides. It is, therefore, critical to understand current public perspectives on nanopesticides among both pesticide users and food consumers, thereby helping industries and governments assess the development trends of nanopesticides and make relevant strategies for production and regulations in the next stage.

## 4. Conclusions

This study combined socioeconomic and technological aspects to evaluate factors that affect public willingness-to-pay (WTP) for nanopesticides and public perceptions from both pesticide users and food consumers perspectives. The findings provide key information for industries and governments to improve marketing strategies and regulations for the large-scale future application of nanopesticides, thus ensuring crop production for global food security and maintaining agricultural sustainability.

As this study demonstrated, nanopesticides were highly accepted by pesticide users, and 97.4% were willing to spend money on them. The main price range (%) pesticide users were willing to pay for nanopesticides was 25–40% higher than that for conventional pesticides. The experience of applying pesticides had a greater influence on the WTP for nanopesticides than the other variables. Familiarity, trust in industries, attitude, and income were also positive and significant determinants of WTP for nanopesticides. The general public’s familiarity level with nanopesticides was low. Nevertheless, both pesticide users and food consumers supported the future development of nanopesticides quite strongly. Most of the participants agreed that nanopesticides must include labels indicating that the product contains nanocomponents. Pesticide users generally trusted governments and industries, while a few food consumers had neutral or distrust levels in industries for selling and production.

Based on our findings, we suggest that governments should take label requirements into account when developing regulations. The related knowledge of nanopesticides should also be provided to the public via media, lectures, and training programs. In addition, governments should take responsibilities for optimizing relevant regulatory frameworks, such as the standard code of nanopesticides for entering markets, the legality of industries, and the use and recycling of nanopesticides.

The current study is also subject to certain limitations, as survey results were based on Chinese samples. It would be important to conduct local studies in different countries with larger sample sizes, since public responses may vary with cultures and traditions [64]. Moreover, although we measured public WTP for nanopesticides, a divergence may exist between intentions and actual purchasing behaviors [65]; hypothetical WTP values were typically higher than the real WTP values [66]. Compared with the survey scenarios, people may be more frugal in real life as a result of budget constraints, policy implications, etc. [32]. Furthermore, the current study only investigated the factors influencing pesticide users’ WTP for nanopesticides. It would also be essential to identify the factors influencing food consumers’ WTP for nanopesticide-engaged foods. The social acceptance and successful application of nanoproducts depend on complex aspects [63]. The associated considerations of nanopesticides, such as cost assessment, environmental impact, risks to human health, and ethical issues, still need to be addressed more comprehensively in future research.

## Figures and Tables

**Table 1:** Overview and measurements of the variables and descriptive statistics of 232 pesticide users.

Dependent Variable	Description and Measurement	Mean	Median	Standard Deviation	Min	Max
Decision to spend money on nanopesticides at a lower price	No = 0, Yes = 1	0.97	1	0.16	0	1
Price ranges of willingness-to-pay	The percentage that consumers were willing to pay higher than conventional pesticides for nanopesticides: (−100%, 0) = 1, [0, 10%) = 2, [10%, 25%) = 3, [25%, 40%) = 4, [40%, 50%) = 5, [50%, 75%) = 6, [75%, 100%) = 7, [100%, 130%) = 8, ≥130% = 9	4.95	4	2.56	1	9
**Independent Variable**	**Description and Measurement**	**Mean**	**Median**	**Standard Deviation**	**Min**	**Max**
Gender	Female = 1, Male = 0	0.17	0	0.38	0	1
Age	Full year of age	45.53	46	9.62	25	75
Years of education	Seven categories: uneducated = 0, primary school = 6, middle school = 9, high school/professional high school/technical school/secondary school = 12, junior college = 15, undergraduate education = 16, postgraduate education = 19 Unit: years	11.08	12	2.72	6	16
Total household income in 2019	Unit: 100,000 RMB (approximately 15,385 USD)	4.06	1.3	15.69	0.07	222
Experience of applying pesticides	Unit: years	15.57	12.5	10.89	0	52
Familiarity with nanopesticides	Completely unfamiliar = 1, A little unfamiliar = 2, General = 3, Quite familiar = 4, Very familiar = 5	2.64	3	1.07	1	5
Attitude toward the future development of nanopesticides	Very opposed = 1, A little opposed = 2, Neutral = 3, Quite supportive = 4, Very supportive = 5	4.00	4	0.69	2	5
Labeling preference	Do you agree that the product label of nanopesticides must indicate that it contains nano-components? Completely disagree = 1, A little disagree = 2, Neutral = 3, Quite agree = 4, Strongly agree = 5	4.22	4	0.68	3	5
Social trust	Completely distrust = 1, A little distrust = 2, General = 3, Quite trust = 4, Strongly trust = 5					
Trust in governments	Do you trust that governments could supervise the safety risks of nanopesticides?	4.13	4	0.78	1	5
Trust in industries	Do you trust that manufactures and retailers could produce and sell nanopesticides legally?	3.86	4	0.79	1	5

**Table 2:** Interval regression model for evaluating factors influencing pesticide users’ WTP for nanopesticides, and OLS and ordered logistic models for testing the robustness of the interval regression model.

Variable	Interval Regression Model		OLS Model		Ordered Logistic Model	
	Coefficient	Robust Standard Error	Coefficient	Robust Standard Error	Coefficient	Robust Standard Error
Gender	−3.61	10.99	−3.56	9.00	−0.41	0.41
Age	−0.10	0.54	−0.08	0.47	−0.02	0.02
Years of education	1.97	1.38	1.70	1.18	0.12 *	0.05
Experience of applying pesticides	−2.77 *	1.08	−2.27 *	0.89	−0.08 *	0.04
Quadratic term of experience of applying pesticides	0.05 *	0.02	0.04 *	0.02	0.00 *	0.00
Income	1.39 *	0.57	1.15 **	0.39	0.05 **	0.02
Familiarity with nanopesticides	11.08 **	3.39	8.55 **	2.76	0.46 **	0.12
Attitude toward nanopesticides	13.38 *	6.65	13.70 *	5.87	0.61 *	0.26
Trust in governments	−7.64	5.60	−6.37	4.84	−0.27	0.22
Trust in industries	13.83 *	5.52	10.52 *	4.59	0.29	0.19
Labeling preference	0.04	6.26	−0.39	5.44	0.20	0.27
Constant	−43.22	38.93	−36.62	33.08		
Wald test	Chi-square = 57.34; *p* = 0.00					
VIF ^†^	Mean = 1.58					
Numbers of observations	226		226		226	

Notes: ** and * indicate significance at *p* < 0.01 and *p* < 0.05 levels, respectively. The unit of the coefficients is percentage points. ^†^ The mean value of VIF (variance inflation factor) was smaller than 2, indicating no multicollinearity between the independent variables in the regression model.

## Data Availability

The data presented in this study are available on request from the corresponding author. The data are not publicly available due to privacy.

## Appendix A

Appendix A contains the distribution of sampling sites in the western, middle, and eastern parts of China (Figure A1), and the sequence of questions about the willingness-to-pay for nanopesticides (Figure A2). The theory underlying the Heckman model and formulas of interval regression model are also provided in Appendix A. Table A1 presents the Heckman model for testing sample selection bias. Table A2 and Table A3 provide the standardized beta coefficients of influencing factors, and different pesticide user profiles’ willingness-to-pay for nanopesticides, respectively.

### Appendix A.1. Theory of Heckman Model

Sample selection bias may arise when values of dependent variables are missing or unobserved, caused by another process (e.g., self-selection by individuals or data units investigated, sample selection decisions by analysts or data processers) [46,47]. For example, if the appearance of outcome variable $y_{i}$ depends on a selection variable $z_{i}$, such incidental truncation may result in a missing data problem of $y_{i}$ and biased coefficient estimation using standard regression techniques (e.g., OLS). In order to resolve this potential bias, the Heckman model was introduced and assumed the underlying two-stage relationship [48]:

The selection equation is shown below:

(A1) $$z_{i}^{*}=\alpha_{i}\gamma +u_{i}\;z_{i}=\{\begin{array}{c} 1\;\;\;\;\;\;\;if\;z_{i}^{*}>0\\ 0\;\;\;\;\;\;\;if\;z_{i}^{*}\leq 0 \end{array}$$

The outcome equation is as follows:

(A2) $$y_{i}=\{\begin{array}{cc} x_{i}\beta +\varepsilon_{i} & if\;z_{i}=1\\ unobserved & if\;z_{i}=0 \end{array}$$

where $x_{i}$ are covariates that affect the outcome and $\alpha_{i}$ are covariates that affect selection, $\varepsilon_{i}\sim N\left(0,\sigma^{2}\right)$, $u_{i}\sim N\left(0,1\right)$, $corr\left(\varepsilon_{i},u_{i}\right)=\rho$.

The log likelihood for observation i is $\ln L_{i}$:

(A3) $$\ln L_{i}=\{\begin{array}{cc} \omega_{i}\ln\Phi \left\{\frac{\alpha_{i}\gamma +\left(y_{i}-\;x_{i}\beta \right)\rho /\sigma }{\sqrt{1-\rho^{2}}}\right\}-\frac{\omega_{i}}{2}{\left(\frac{y_{i}-\;x_{i}\beta }{\sigma }\right)}^{2}-\omega_{i}\ln\left(\sqrt{2\pi }\sigma \right)\;\;\;\;\;\;\;\;\;\; & y_{i}\mathrm{observed}\\ \omega_{i}\ln\Phi \left(-\alpha_{i}\gamma \right) & y_{i}\mathrm{unobserved} \end{array}$$

where Φ is the standard cumulative normal distribution and $\omega_{i}$ is an optional weight for observation i.

The first stage is to determine whether an observation in an overall population appears in the final representative samples, and the second stage is to model the relationship between the dependent and independent variables in the final selected samples [46]. With maximum likelihood estimation in the Heckman model, rho (ρ; the correlation between error terms in the selection and outcome equations) could be examined to indicate whether or not sample selection bias exists [46]. If $\varepsilon_{i}$ and $u_{i}$ are correlated, traditional techniques (e.g., OLS) would report biased β estimation. In this situation, the results of the Heckman model could be consistent and asymptotically efficient estimates by correcting selection bias [48]. Otherwise, traditional regression methods could generate efficient estimates by using selected samples. Since the formulas above are appropriate for general-type data, the formulas extended for the interval data of our study could be found in the command *eintreg* of Stata manual Release 15 [67].

### Appendix A.2. Formulas of Interval Regression Model

The equation of the interval regression model [48] is as follows:

(A4) $$Y_{i}^{*}=X_{i}\beta +\varepsilon_{i}$$

where $Y_{i}^{*}$ is a continuous outcome for the *i*th observation with covariates $X_{i}$ and corresponding coefficients β. ε is the error term that is assumed to be mean zero and normally distributed; ε∼*N* (0, *σ*^2^).

If observation *i* ∈ *C* (not censored), we observe $Y_{i}^{*}$ as the point data. If observation *i* ∈ *L* (left-censored), the unobserved $Y_{i}^{*}$ is in the interval (–∞, $Y_{i2}$]. The likelihood contribution is as follows:

(A5) $$\mathrm{Pr}\left(Y_{i}^{*}\leq Y_{i2}\right)=\mathrm{Pr}\left(X_{i}\beta +\varepsilon_{i}\leq Y_{i2}\right)$$

If *i* ∈ *I* (interval-censored), the unobserved $Y_{i}^{*}$ is in the interval [$Y_{i1}$, $Y_{i2}$]. The likelihood contribution is as follows:

(A6) $$\mathrm{Pr}\left(Y_{i1}\leq Y_{i}^{*}\leq Y_{i2}\right)=\mathrm{Pr}\left(Y_{i1}\leq X_{i}\beta +\varepsilon_{i}\leq Y_{i2}\right)$$

If *i* ∈ *R* (right-censored), the unobserved $Y_{i}^{*}$ is in the interval [$Y_{i1}$, +∞). The likelihood contribution is shown below:

(A7) $$\mathrm{Pr}\left(Y_{i1}\leq Y_{i}^{*}\right)=\mathrm{Pr}\left(Y_{i1}\leq X_{i}\beta +\varepsilon_{i}\right)$$

The total loglikelihood function is given as:

(A8) $$\ln L=-\frac{1}{2}\underset{i\in C}{\sum }\omega_{i}\left\{{\left(\frac{Y_{i}^{*}-X_{i}\beta }{\sigma }\right)}^{2}+\log2{\pi \sigma }^{2}\right\}\;+\underset{i\in L}{\sum }\omega_{i}\log\Phi \left(\frac{Y_{i2}-X_{i}\beta }{\sigma }\right)\;+\underset{i\in I}{\sum }\omega_{i}\log\left\{\Phi \left(\frac{Y_{i2}-X_{i}\beta }{\sigma }\right)-\Phi \left(\frac{Y_{i1}-X_{i}\beta }{\sigma }\right)\right\}\;+\underset{i\in R}{\sum }\omega_{i}\log\left\{1-\Phi \left(\frac{Y_{i1}-X_{i}\beta }{\sigma }\right)\right\}$$

Note that Φ is the cumulative standard normal distribution and $\omega_{i}$ is the weight for the *i*th observation [48]. The coefficients could be estimated by maximizing the value of the loglikelihood function lnL.

**Table A1:** Heckman model for testing sample selection bias.

Variable	Heckman Model	
	Coefficient	Robust Standard Error
Gender	−4.46	10.94
Age	−0.12	0.54
Years of education	2.15	1.39
Experience of applying pesticides	−2.88 **	1.09
Quadratic term of experience of applying pesticides	0.05 *	0.02
Income	1.42 *	0.57
Familiarity with nanopesticides	11.19 **	3.40
Attitude toward nanopesticides	13.79 *	6.67
Trust in governments	−7.96	5.66
Trust in industries	13.57 *	5.53
Labeling preference	−0.65	6.29
Constant	−39.45	38.97
rho	−0.42	0.33
Wald test	Chi-square = 57.95; *p* = 0.00	
VIF	Mean = 1.59	
Numbers of observations	232	

Notes: ** and * indicate significance at the *p* < 0.01 and *p* < 0.05 levels, respectively. The unit of the coefficients is percentage points.

**Table A2:** Standardized beta coefficients of influencing factors.

Variable	Standardized Beta Coefficient
Experience of applying pesticides	−0.58 *
Familiarity with nanopesticides	0.23 **
Trust in industries	0.21 *
Attitude toward nanopesticides	0.18 *
Income	0.17 *
Trust in governments	−0.12
Years of education	0.10
Gender	−0.03
Age	−0.02
Labeling preference	0.00

Note: ** and * indicate significance at the *p* < 0.01 and *p* < 0.05 levels, respectively.

**Table A3:** Different pesticide user profiles’ willingness-to-pay for nanopesticides.

Experience of Applying Pesticides	Familiarity with Nanopesticides	Trust in Industries	The Percentage That Pesticide Users Were Willing to Pay Higher for Nanopesticides than That for Conventional Pesticides
23	2	1	−1.00%
13	2	1	8.27%
23	1	3	15.57%
5	2	1	23.04%
13	1	3	24.84%
23	2	3	26.66%
13	2	3	35.92%
23	3	3	37.74%
23	2	4	40.48%
13	3	3	47.00%
13	2	4	49.75%
5	2	3	50.69%
23	3	4	51.56%
13	3	4	60.83%
5	3	3	61.77%
5	2	4	64.52%
5	3	4	75.60%
5	4	5	100.51%
5	5	5	111.59%

## Appendix B. Questionnaire of Public Perceptions and Willingness-to-Pay for Nanopesticides

1. What is your gender?
  1 = Female, 0 = Male

2. What is your full year of age?
3. What is your educational level?
  1 = uneducated, 2 = primary school, 3 = middle school, 4 = high school, 5 = professional high school/technical school, 6 = secondary school, 7 = junior college, 8 = undergraduate education, 9 = postgraduate education

4. What is your total household income in 2019?
5. How are you familiar with nanopesticides?
  1 = Completely unfamiliar, 2 = A little unfamiliar, 3 = General, 4 = Quite familiar, 5 = Very familiar

Nanopesticides are composed of nanomaterials (1–100 nm, 1 nm = 1/10^9^ m) with broad-spectrum insecticidal, fungicidal, or herbicidal properties [15]. Compared with conventional chemical pesticides, nanopesticides could improve efficacy 24~150% [15], prolong effective time period [68], lower application rates [12], and increase yields [15]. In addition, nanopesticides could have less residuals and decrease environmental burden [68]. The potential negative impacts on human health and environment are low, but there is still a lack of comprehensive evaluation [69].

6. What is your attitude toward the future development of nanopesticides?
  1 = Completely opposed, 2 = A little opposed, 3 = Neutral, 4 = Quite supportive, 5 = Very supportive

7. Do you agree that the product label of nanopesticides must indicate it contains nano- components?
  1 = Completely disagree, 2 = A little disagree, 3 = Neutral, 4 = Quite agree, 5 = Strongly agree

8. Do you trust that governments could supervise the safety risks of nanopesticides?
  1 = Completely distrust, 2 = A little distrust, 3 = General, 4 = Quite trust, 5 = Strongly trust

9. Do you trust that industries (manufactures and retailers) could produce and sell nanopesticides legally?
  1 = Completely distrust, 2 = A little distrust, 3 = General, 4 = Quite trust, 5 = Strongly trust

10. Do you plant grain, vegetables, and fruit that need pesticides?
  1 = Yes, 0 = No
  (If yes, please answer the following questions; If no, please quit the survey)

11. You have ___ years of experience in applying pesticides.
12. The price ranges of willingness-to-pay for nanopesticides:

a. Would you be willing to purchase nanopesticides if the price is lower than conventional pesticides?	1 = Yes (continue with Question b); 2 = No (stop answering and quit the survey)
b. Would you be willing to purchase nanopesticides if the price is as the same as conventional pesticides?	1 = Yes (continue with Question c); 2 = No (stop answering and quit the survey)
c. Would you be willing to purchase nanopesticides if the price is 50% higher than conventional pesticides?	1 = Yes (skip to Question d); 2 = No (skip to Question e)
d. Would you be willing to purchase nanopesticides if the price is 100% higher than conventional pesticides?	1 = Yes (skip to Question d-1); 2 = No (skip to Question d-2)
d-1. Would you be willing to purchase nanopesticides if the price is 130% higher than conventional pesticides?	1 = Yes; 2 = No
d-2. Would you be willing to purchase nanopesticides if the price is 75% higher than conventional pesticides?	1 = Yes; 2 = No
e. Would you be willing to purchase nanopesticides if the price is 25% higher than conventional pesticides?	1 = Yes (skip to Question e-1); 2 = No (skip to Question e-2)
e-1. Would you be willing to purchase nanopesticides if the price is 40% higher than conventional pesticides?	1 = Yes; 2 = No
e-2. Would you be willing to purchase nanopesticides if the price is 10% higher than conventional pesticides?	1 = Yes; 2 = No
